# Prognostic significance of clinicopathological factors in early breast cancer: 20 years of follow-up in a single-center analysis

**DOI:** 10.18632/oncotarget.18526

**Published:** 2017-06-16

**Authors:** Valentina Cocciolone, Katia Cannita, Maria Letizia Calandrella, Enrico Ricevuto, Paola Lanfiuti Baldi, Tina Sidoni, Azzurra Irelli, Stefania Paradisi, Laura Pizzorno, Valter Resta, Alberto Bafile, Edoardo Alesse, Alessandra Tessitore, Corrado Ficorella

**Affiliations:** ^1^ Medical Oncology, S. Salvatore Hospital, University of L'Aquila, Via Vetoio, 67100 L'Aquila, Italy; ^2^ Department of Biotechnological and Applied Clinical Sciences, University of L'Aquila, Via Vetoio, 67100 L'Aquila, Italy; ^3^ Oncology Network ASL1 Abruzzo, UOSD Oncology Territorial Care, S. Salvatore Hospital, University of L'Aquila, Via Vetoio, 67100 L'Aquila, Italy; ^4^ Breast Unit, S. Salvatore Hospital, L'Aquila, Via Vetoio, 67100 L'Aquila, Italy

**Keywords:** clinicopathological prognostic factors, early breast cancer, clinical practice, survival, 20-year follow-up

## Abstract

**Background:**

To quantify the effect of traditional prognostic factors [nodal status, estrogen-receptor (ER), progesterone-receptor (PR), human epidermal growth factor receptor 2 (HER2)] on long-term outcome of patients with early breast cancer (EBC), treated in clinical practice over a period of about twenty years.

**Results:**

1198 consecutive patients were identified. Median DFS (disease-free survival): ER+/PR±/HER2−, 165 months (mo) if node-negative (N0) and 114mo if node-positive (N+) (*p* < 0.001); triple-negative (TN), 109mo if N0 and 65mo if N+ (p 0.144); ER+/PR±/HER2+ in patients not-treated with adjuvant trastuzumab (T−), not reached if N0 and 114mo if N+ (p 0.297); ER+/PR±/HER2+ in patients treated with trastuzumab (T+), 95mo if N0 and 85mo if N+ (p 0.615); ER−/PR−/HER2+ T−, not reached if N0 and 26mo if N+ (p 0.279); ER−/PR−/HER2+ T+, not reached if N0 and 66mo if N+ (p 0.014). Median OS (overall survival): ER+/ PR±/HER2−, 166mo if N0 and 144mo if N+ (p 0.028); TN, 158mo if N0 and 96mo if N+ (p 0.384); ER+/PR±/HER2+ T−, not reached if N0 and 157mo if N+ (p 0.475), ER+/PR±/HER2+ T+, not reached if N0 and 106mo if N+ (p 0.436); ER−/PR−/HER2+ T−, not reached if N0 and 34mo if N+ (p 0.273); ER−/PR−/HER2+ T+, not reached neither if N0 nor if N+ (p 0.094).

**Materials and Methods:**

Disease-free survival (DFS) and overall survival (OS) were evaluated according to tumor characteristics, based on information retrospectively retrieved from patients’ medical records.

**Conclusions:**

Pathological tumor characteristics and nodal status still represent useful tools in treatment selection and follow-up decision making of EBC patients in clinical practice.

## INTRODUCTION

Accounting for 23% of the total new cases of cancer and 14% of the total cancer deaths, breast cancer (BC) is the most frequently diagnosed cancer in women worldwide and the second leading cause of cancer-related mortality [1]. The overall 5-year relative survival rate for BC patients has improved from 75.1% between 1975 and 1977 to 90.0% from 2001 to 2007, mainly thanks to improvements in treatment and earlier diagnosis due to the widespread use of mammography [2]. The 5-year relative survival rate for women diagnosed with localized BC is approximately 98%, but survival declines to about 84% for regional involvement and 23% for distant disease [3]. Even if BC recurrence usually occurs within the first 5 years after diagnosis, relapse may also happen after 5 years: in a retrospective analysis evaluating 2838 patients receiving adjuvant therapy, who remained disease free for 5 years, the 5-year residual risk of recurrence was 7%, 11% and 13% for stage I, II and III BC, respectively [4]. The risk of metastases and death increases with both BC size at diagnosis and number of axillary lymph nodes involved [5–8]. Tumor grade, hormone receptor status and human epidermal growth factor receptor 2 (HER2) status significantly influence survival [9–11]. In more recent years, (neo-)adjuvant systemic treatment for BC is applied more often and has considerably improved. The introduction of trastuzumab, for instance, significantly increased both short term and long term prognosis in HER2-positive BC patients [12, 13]. Estrogen receptor (ER), progesterone-receptor (PR) and HER2, routinely available in BC tissue samples and recorded in cancer registries and patients’ medical records, are useful tools for therapeutic decision making and could be considered reliable surrogates for the more expensive molecular subtyping [14].

To quantify the effect of traditional prognostic factors, both long term and in the current era, on outcome of BC patients, we performed a single-center, retrospective survival analysis of women with early invasive BC treated in clinical practice at our Department, to determine their disease-free survival (DFS) and overall survival (OS) and to design proper follow-up strategies according to risk of recurrence.

## RESULTS

### Characteristics of patients and tumors and treatment administered to study population

Overall, we retrospectively identified 1198 consecutive, early BC patients treated in clinical practice at Medical Oncology Department, San Salvatore Hospital, L'Aquila, Italy, between June 1992 and December 2013.

Main baseline patient and tumor characteristics are reported in Table 1. Specifically, 55.3% of all tumors had a diameter ≤ 2 cm (T1) and 52.2% did not have lymph nodes involvement at the time of surgery (N0). ER+/PR±/HER2− tumors accounted for 58.4% of cases, ER−/PR−/HER2− for 10.3%, ER+/PR±/HER2+ for 9.1% and ER−/PR−/HER2+ for 6.3%, while for 15.9% of samples the IHC pattern was not fully available.

**Table 1:** Descriptive characteristics of the study population (*N* = 1198) and treatment administered

Characteristics	*n* (%)
**Age (years)**	
median	55
range	24–83
**Histology**	
ductal	846 (71.2)
lobular	257 (21.6)
other	86 (7.2)
**Tumor size**	
T1	663 (55.3)
T2	402 (33.6)
T3	44 (3.7)
T4	31 (2.6)
unknown	58 (4.8)
**Nodal status**	
N0	625 (52.2)
N1	355 (29.6)
N ≥ 2	148 (12.4)
unknown	70 (5.8)
**Grading**	
G1	130 (10.8)
G2	364 (30.4)
G3	569 (47.5)
unknown	135 (11.3)
**Hormone receptor/HER2 status (second generation, *N* = 1096)**	
ER+/PR±/HER2−	640 (58.4)
ER−/PR−/HER2−	113 (10.3)
ER+/PR±/HER2+	100 (9.1)
ER−/PR−/HER2+	69 (6.3)
not fully available	174 (15.9)
**Surgery**	
conservative	875 (73)
radical	323 (27)
**Chemotherapy**	
yes	881 (73.5)
anthracyclines-based	393 (32.8)
taxanes-based	138 (11.5)
anthracyclines and taxanes-based	286 (23.9)
not anthracyclines and not taxanes-based	64 (5.3)
not	317 (26.5)
**Endocrine therapy**	
yes	941 (78.5)
post-menopausal	
tamoxifen	134 (11)*
aromatase-inhibitor	293 (24.5)
tamoxifen → aromatase inhibitor	101 (8)
pre-menopausal	
tamoxifen	58 (5)
tamoxifen + LH-RH analogue	209 (17.5)
aromatase-inhibitor + LH-RH analogue	54 (4.5)
tamoxifen → aromatase inhibitor (+ LH-RH analogue)	92 (8)
not	257 (21.5)
**Radiotherapy**	
yes	919 (76.7)
not	279 (23.3)

*This group includes post-menopausal patients treated with endocrine therapy before the introduction of aromatase inhibitors in clinical practice in Italy (about 1997).

Data on adjuvant treatment administered to study population are summarized in Table 1.

To note, 73% of patients underwent conservative surgery, 73.5% received chemotherapy (anthracyclines-based, 32.8%; taxanes-based, 11.5%; anthracyclines and taxanes-based, 23.9%; not anthracyclines and not taxanes-based, 5.3%) and 78.5% endocrine therapy.

### Survival analysis

Median follow-up for the whole patient population was 93 months, being 196 months for the first generation and 85 months for the second generation. Overall, we observed 240 disease recurrences (20%), represented by loco-regional relapse in 95 patients (8%), contralateral tumor in 31 patients (2.5%) and distant metastatic spread in 114 patients (9.5%). At the time of data cut-off, 1092 patients (91.1%) were still alive and 106 (8.9%) had died. The overall median DFS was 132 months (95% Confidence Interval (CI), 3.90 to 4.03); the 1-year, 5-year and 10-year DFS rates were 91%, 80% and 52%, respectively. The overall median OS was 162 months (95% CI, 4.63 to 4.82); the 1-year, 5-year and 10-year OS rates were 96%, 82.5% and 60.5%, respectively.

The overall median DFS was 92 months (95% CI, 9.31 to 10.10) and 137 months (95% CI, 4.46 to 4.64) for the first and second generation, respectively; the overall median OS was 127 months (95% CI, 9.33 to 10.10) and 166 months (95% CI, 5.90 to 6.20), respectively.

### Impact of nodal status on survival

According to the nodal status, patients were classified as N0 (no positive lymph nodes), N1-3 (1–3 positive lymph nodes), N4+ (≥ 4 positive lymph nodes). For 70 patients the nodal status was not available.

**Figure 1:**
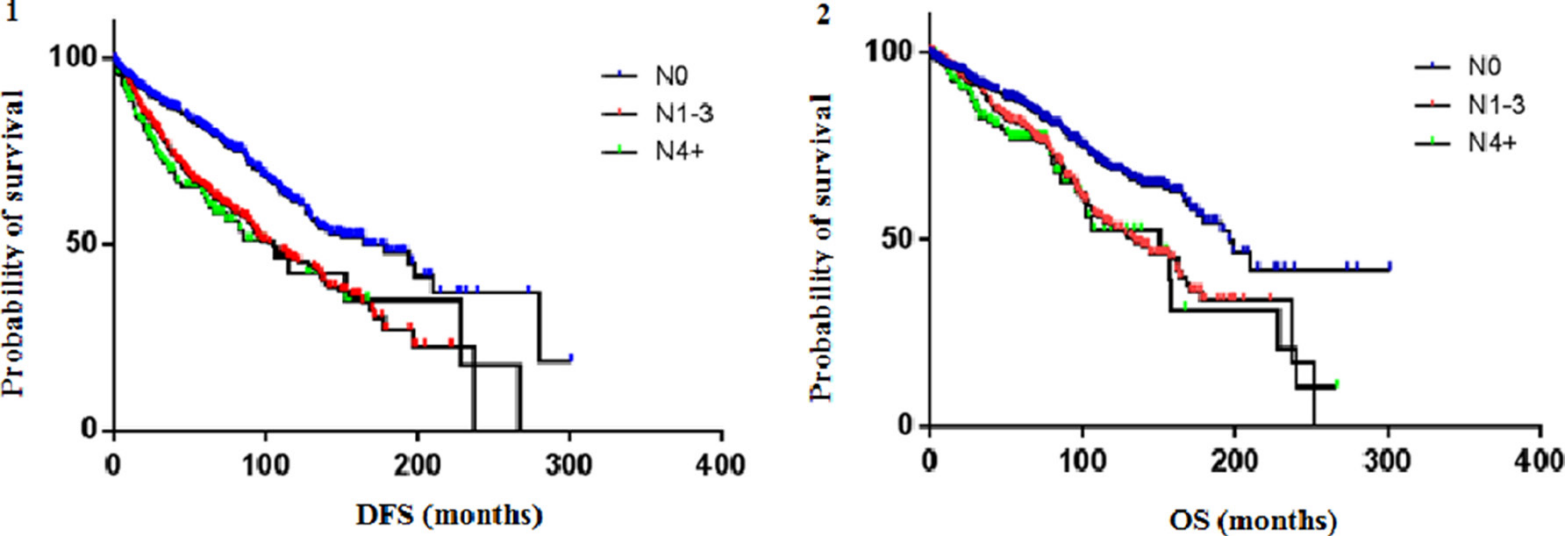
DFS (**1**) and OS (**2**) according to nodal status. The median DFS was 165 months (95% CI 6.55 to 6.94), 105 months (95% CI 6.03 to 6.36) and 106 months (95% CI 12.50 to 13.60) for the N0, N1-3 and N4+ subgroups, respectively. The median OS was 196 months (95% CI 9.44 to 10.26), 134 months (95% CI 6.64 to 7.04) and 151 months (95% CI 15.62 to 17.34) for the N0, N1-3 and N4+ subgroups, respectively.

The median DFS was 165 months (95% CI 6.55 to 6.94), 105 months (95% CI 6.03 to 6.36) and 106 months (95% CI 12.50 to 13.60) for the N0, N1-3 and N4+ subgroups, respectively. At 1 year from diagnosis, 94% of N0 patients was still disease-free, compared to 91% of N1-3 and 88% of N4+ patients; at 5 years, DFS rates were 81%, 65.2% and 63%, respectively; at 10 years, DFS rates were 61.5%, 46% and 42%, respectively. The median OS was 196 months (95% CI 9.44 to 10.26), 134 months (95% CI 6.64 to 7.04) and 151 months (95% CI 15.62 to 17.34) for the N0, N1-3 and N4+ subgroups, respectively. At 1 year from diagnosis, 96% of N0 patients, 96% of N1-3 and 91% of N4+ patients was still alive; at 5 years, OS rates were 86.4%, 80.4% and 77%, respectively; at 10 years, OS rates were 68.5%, 53.4% and 52.4%, respectively. Figure 1 shows Kaplan-Meier estimates of DFS and OS according to nodal status. DFS and OS rates according to nodal status are shown in Table 2.

**Table 2:** Survival rates according to nodal status

	Median DFS (months)	% DFS			Median OS(months)	% OS		
		2y	5y	10y		2y	5y	10y
**Overall *n* = 1128**	132	85.6	80	52	162	92.8	82.5	60.5
**N0 *n* = 625**	165	89.4	81	61.5	196	93.4	86.4	68.5
**N1-3 *n* = 355**	105	83.6	65.2	46	134	92.3	80.4	53.4
**N4+ *n* = 148**	106	78.3	63	42	151	90	77	52.4

**Figure 2:**
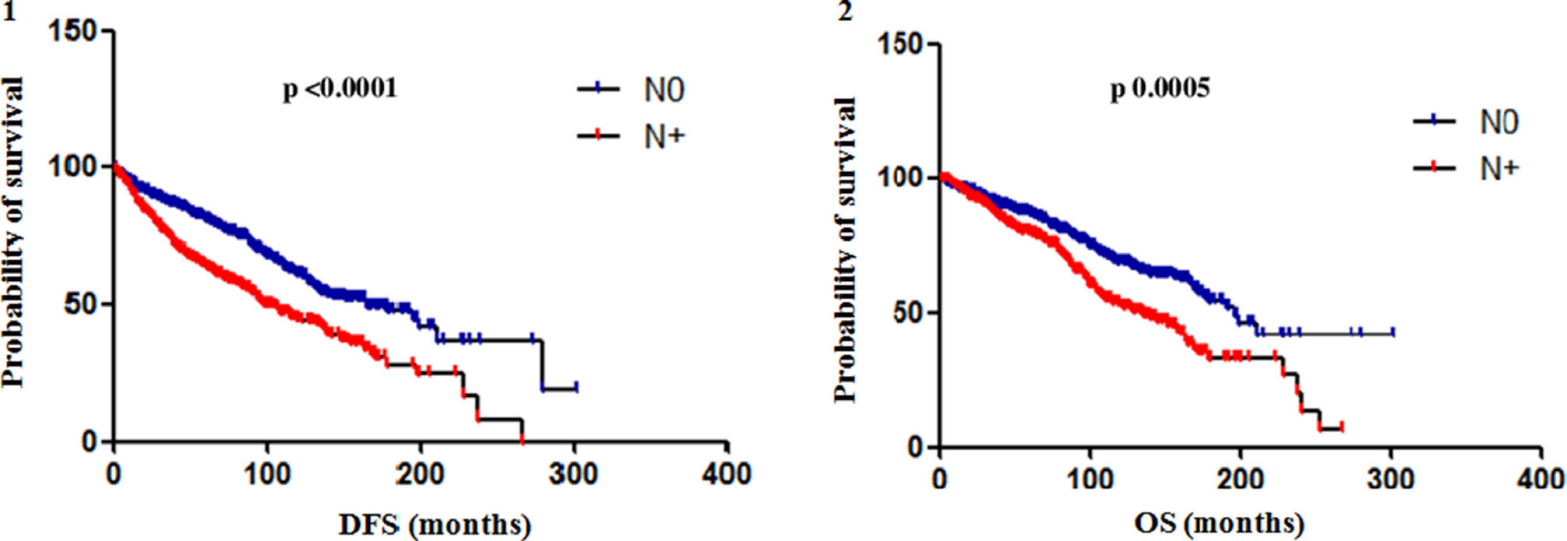
Difference in DFS (**1**) and OS (**2**) between N0 and N+ subgroups. The difference in median DFS between N0 and N+ (data on N1-3 and N4+ subgroups were collected together in the category N+) subgroups was statistically significant (95% CI 0.44 to 0.66, HR 0.54, *p* < 0.0001) as well as the difference in median OS (95% CI 0.47 to 0.74, HR 0.59, p 0.0005).

Both median DFS and OS were not significantly different between N1-3 and N4+ subgroups (DFS: 95% CI 0.64 to 1.22, Hazard Ratio (HR) 0.89, p 0.489; OS: 95% CI 0.61 to 1.28, HR 0.89, p 0.542). So, data on N1-3 and N4+ subgroups were collected together in the category N+ and compared to N0 subgroup. Median DFS of N+ patients was 102 months and median OS 134 months. The difference in median DFS between N0 and N+ subgroups was statistically significant (95% CI 0.44 to 0.66, HR 0.54, *p* <0.0001) as well as the difference in median OS (95% CI 0.47 to 0.74, HR 0.59, p 0.0005) (Figure 2).

### Impact of hormone receptor/HER2 status on survival

Survival analysis according to the tumor IHC pattern was performed only for patients of the second generation (HER2 status evaluated). Anyway, for 174 out of 1096 patients of the second generation the full receptor assessment was not available (*n* = 922).

At median follow-up 85 months, patients with ER+/PR±/HER2- tumors showed a median DFS of 162 months (95% CI, 13.94 to 14.95) and a median OS of 165 months (95% CI, 11.36 to 12.35). Patients with ER−/PR−/HER2- (triple negative, TN) tumors had a median DFS of 92 months (95% CI, 10.16 to 11.02) and a median OS of 158 months (95% CI, 13.78 to 15.60). In particular, in this subgroup, the 1-year and 5-year DFS rates were 86.5% and 60%, respectively, with a plateau in the curve starting from about 100 months from diagnosis (8-year DFS, 40%); the 1-year and 5-year OS rates were 96.4% and 68.7%, respectively.

In the HER2+ subgroup, when both patients treated and not-treated with adjuvant trastuzumab were included, the overall median DFS was 130 months (95% CI, 11.46 to 12.57) and the median OS 157 months (95% CI, 13.97 to 15.56). According to the hormone receptor status, their outcome was separately evaluated: patients with ER+/PR±/HER2+ tumors had a median DFS of 132 months (95% CI, 16.13 to 18.42) and a median OS not reached; patients with ER−/PR−/HER2+ tumors had a median DFS of 91 months (95% CI, 13.95 to 15.82) and a median OS not reached. The analysis of the DFS curves for these subgroups showed that the 2-year and the 5-year DFS rates were 88.7% and 78%, respectively, for patients with ER+/PR±/HER2+ tumors and 72% and 59%, respectively, for patients with ER−/PR−/HER2+ tumors (Table 3).

**Table 3:** Survival rates according to hormone receptor/HER2 status (all patients)

	Median DFS (months)	% DFS			Median OS (months)	% OS		
		2y	5y	10y		2y	5y	10y
**ER+/PR±/HER2− *n* = 640**	162	87.2	76	57.5	165	93	84.5	61
**ER-/PR-/HER2− *n* = 113**	92	75	60	39.3	158	89.7	68.7	55.9
**ER+/PR±/HER2+ *n* = 100**	132	88.7	78	59	not reached	95	86.8	65
**ER-/PR-/HER2+ *n* = 69**	91	72	59	46	not reached	89	65	60

**Figure 3:**
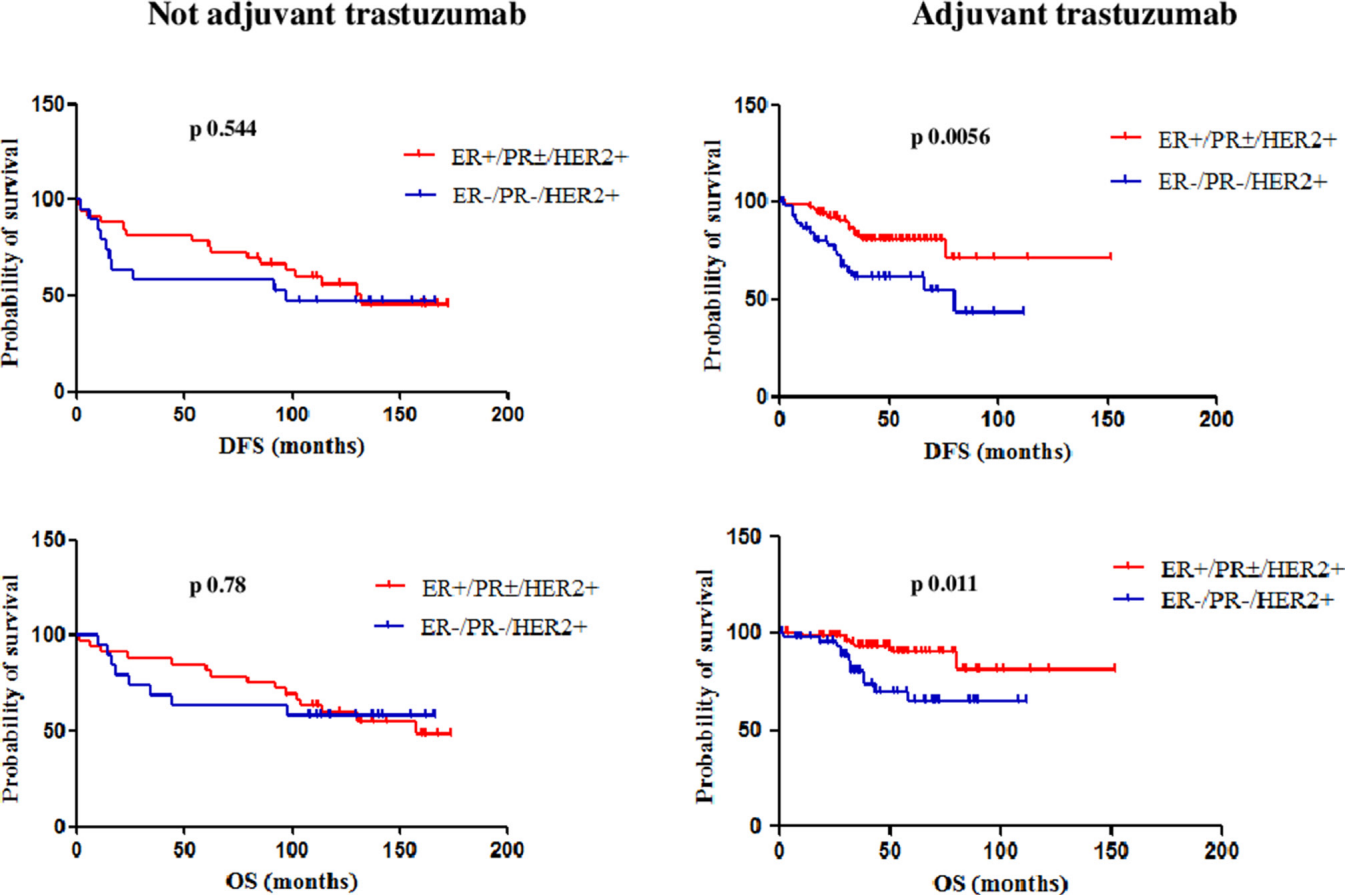
DFS and OS of ER+/PR±/HER2+ and ER-/PR-/HER2+ patients treated and not-treated with adjuvant trastuzumab A statistically significant difference in both DFS and OS was observed between patients with ER+/PR±/HER2+ and ER−/PR−/HER2+ tumors only in the cohort of patients treated with adjuvant trastuzumab.

**Figure 4:**
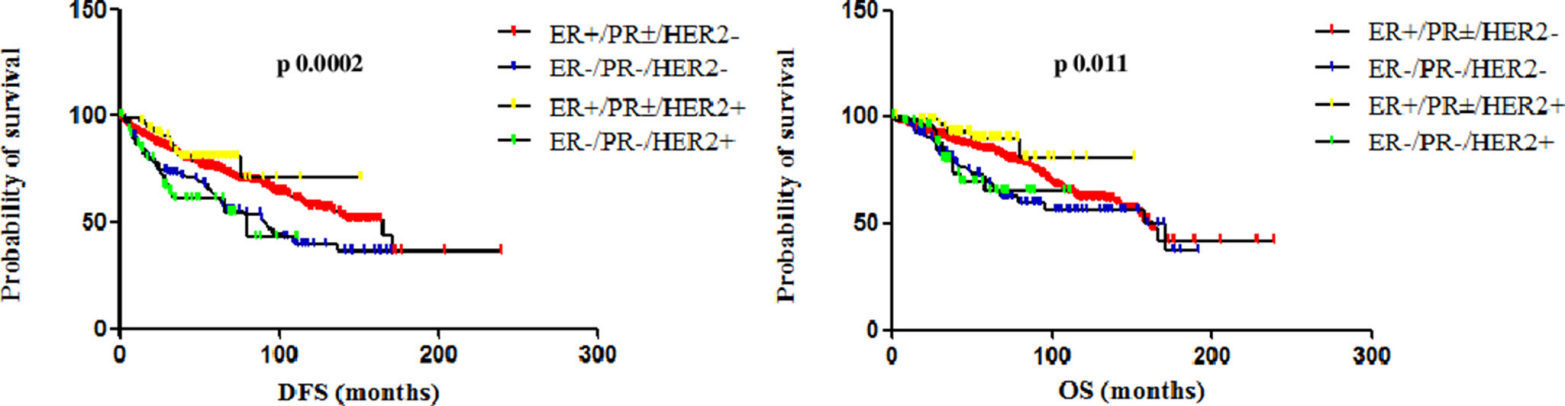
DFS and OS of ER+/PR±/HER2−, ER-/PR-/HER2−, ER+/PR±/HER2+ and ER−/PR−/HER2+ (treated with adjuvant trastuzumab) When including in the HER2+ subgroup only patients treated with adjuvant trastuzumab, patients with ER+/PR±/HER2**−** and ER+/PR±/HER2+ tumors had a significantly better prognosis than those with TN and ER**−**/PR**−**/HER2+ tumors, for both DFS (p 0.0002) and OS (p 0.011).

When data from HER2+ patients treated and not-treated with adjuvant trastuzumab were separately analyzed, a statistically significant difference in both DFS and OS was observed between patients with ER+/PR±/HER2+ and ER−/PR−/HER2+ tumors only in the cohort of patients treated with adjuvant trastuzumab. In this cohort, the median DFS was not reached for the ER+/PR±/HER2+ subgroup and 80 months for the ER−/PR−/HER2+ subgroup (95% CI 0.16 to 0.73, HR 0.34, p 0.0056); the median OS was not reached for both subgroups (95% CI 0.11 to 0.75, HR 0.28, p 0.011). On the other hand, in the cohort of patients not-treated with adjuvant trastuzumab, the median DFS was 132 months for the ER+/PR±/HER2+ subgroup and 97 months for the ER−/PR−/HER2+ subgroup (95% CI 0.34 to 1.76, HR 0.77, p 0.544); the median OS was 157 months for the ER+/PR±/HER2+ subgroup and not reached for the ER−/PR−/HER2+ subgroup (95% CI 0.38 to 2.22, HR 0.93, p 0.78) (Figure 3). Figure 4 shows Kaplan-Meier curves of DFS and OS of all four analyzed subgroups, including in the HER2+ subgroup only patients treated with adjuvant trastuzumab: patients with ER+/PR±/HER2- and ER+/PR±/HER2+ tumors had a significantly better prognosis than those with TN and ER−/PR−/HER2+ tumors, for both DFS (p 0.0002) and OS (p 0.011).

### Impact of the combination of nodal status and hormone receptor/HER2 status on survival

To better define the impact of both nodal status and IHC tumor pattern on outcome, a combined analysis of survival was performed.

ER+/PR±/HER2− tumors occurred at diagnosis with no lymph nodes involvement (N0) in 52.4% of cases (*n* = 335) and with lymph nodes positivity (N+) in 44.2% (*n* = 283); in 3.4% of cases nodal status was not available. TN tumors were N0 at diagnosis in 53.1% of cases (*n* = 60) and N+ in 37.2% (*n* = 42); in 9.7% of cases nodal status was not available. ER+/PR±/HER2+ tumors were N0 at diagnosis in 54% of cases (*n* = 54) and N+ in 34% (*n* = 34); in 12% of cases nodal status was not available. ER−/PR−/HER2+ tumors were N0 at diagnosis in 39.1% of cases (*n* = 27) and N+ in 50.7% (*n* = 35); in 10.2% of cases nodal status was not available. DFS and OS rates according to node and hormone receptor/HER2 status are shown in Table 4.

**Table 4:** Survival rates according to nodal and hormone receptor/HER2 status

		% DFS			% OS			Median follow up (months)
		2y	5y	10y	2y	5y	10y	
**ER+/PR±/HER2−**	**N0**	89	82	65.6	92	87	68	75
	**N+**	85	70	47	92	82	55	71
**ER−/PR−/HER2−**	**N0**	82.8	68	42	91.4	71.8	61.2	93
	**N+**	71.8	56	36.4	87.3	62.2	44.8	89
**ER+/PR±/HER2+**	**N0**	92.5	80.4	57.5	96.2	87	64	73
	**N+**	85	73	49	90.9	87.3	61	74
**ER−/PR−/HER2+**	**N0**	90.7	85	58.5	100	95	83	70
	**N+**	64.3	46	34.4	87	56.5	56.5	65

In the ER+/PR±/HER2- subgroup, median DFS was 165 months and 114 months in case of N0 and N+ tumors, respectively (*p* < 0.001) and median OS 166 months and 144 months, respectively (p 0.028), with statistically significant differences.

In the TN subgroup, median DFS was 109 months and 65 months in case of N0 and N+ tumors, respectively (p 0.144) and median OS 158 months and 96 months, respectively (p 0.384), with not statistically significant differences, but with a trend toward a poorer prognosis in case of nodal involvement.

**Figure 5:**
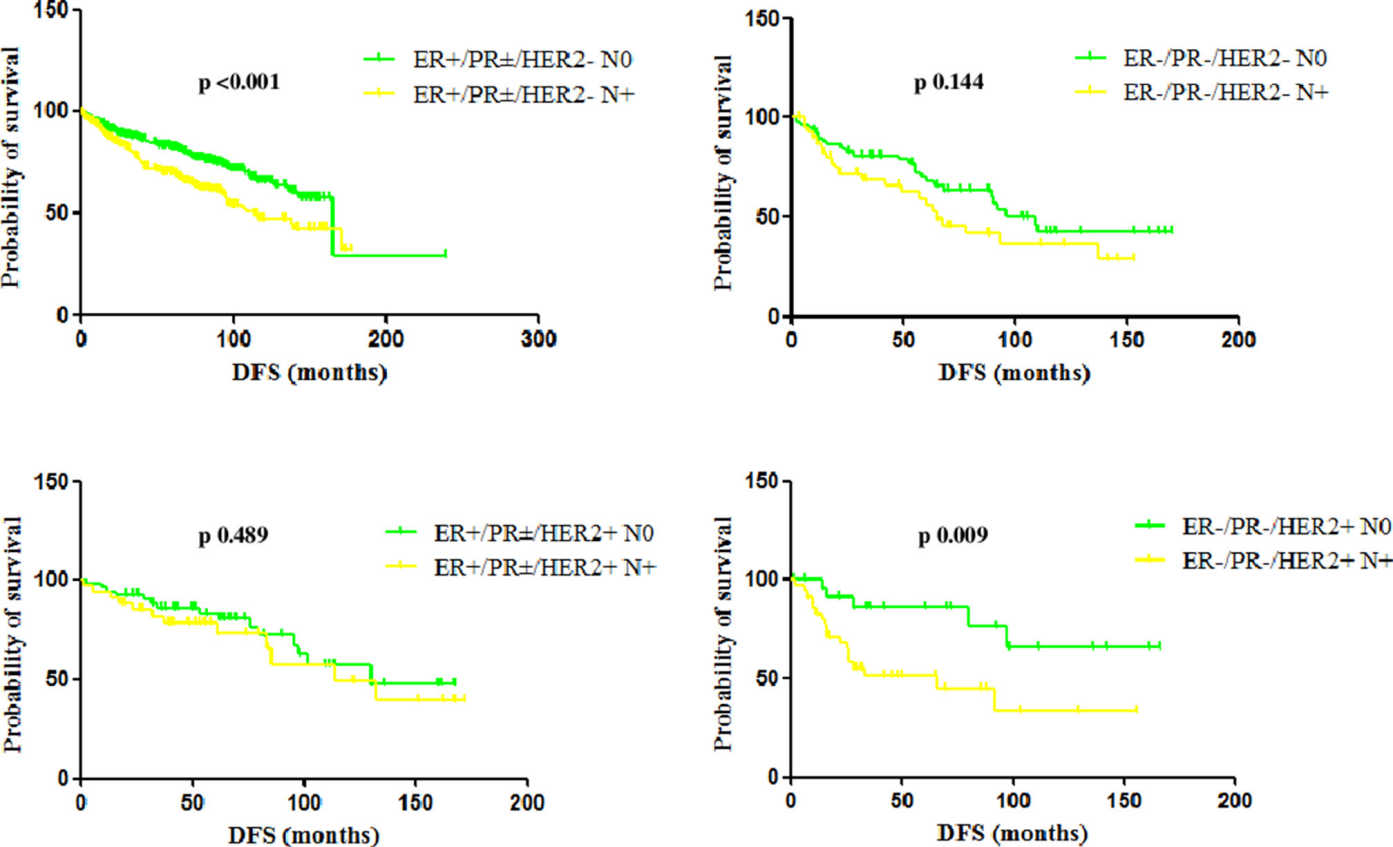
Median DFS according to node and hormone receptor/HER2 status In the ER+/PR±/HER2- subgroup, median DFS was 165 months and 114 months in case of N0 and N+ tumors, respectively (*p* < 0.001). In the TN subgroup, median DFS was 109 months and 65 months in case of N0 and N+ tumors, respectively (p 0.144), with a not statistically significant difference, but with a trend toward a poorer prognosis in case of nodal involvement. Patients with ER+/PR±/HER2+ tumors had a median DFS of 130 months if N0 and 114 months if N+ (p 0.489), while patients with ER−/PR−/HER2+ tumors had a median DFS not reached if N0 and of 66 months if N+ (p 0.009).

In the HER2+ subgroup, a different impact of nodal status can be observed based on the receptor status. Patients with ER+/PR±/HER2+ tumors had a median DFS of 130 months if N0 and 114 months if N+ (p 0.489) and a median OS not reached if N0 and of 157 months if N+ (p 0.876), showing differences not statistically significant according to the nodal status. On the other hand, patients with ER−/PR−/HER2+ tumors had a median DFS not reached if N0 and of 66 months if N+ (p 0.009) and a median OS not reached neither if N0 nor if N+ (p 0.039), with a significant impact of the nodal status on outcome (Table 5). Figure 5 shows Kaplan-Meier curves on DFS according to node and hormone receptor/HER2 status.

**Table 5:** Median DFS and OS according to nodal and hormone receptor/HER2 status

		Median DFS (months)	*p*	Median OS (months)	*p*
**ER+/PR±/HER2−**	**N0**	165	< 0.001	166	0.028
	**N+**	114		144	
**ER−/PR−/HER2-**	**N0**	109	0.144	158	0.384
	**N+**	65		96	
**ER+/PR±/HER2+**	**N0**	130	0.489	not reached	0.876
	**N+**	114		157	
**ER−/PR−/HER2+**	**N0**	not reached	0.009	not reached	0.039
	**N+**	66		not reached	

A separate analysis was performed to assess the impact of nodal status on outcome in the two different subgroups of patients with HER2+ tumors, according to treatment with adjuvant trastuzumab. Patients with ER+/PR±/HER2+ tumors not-treated with trastuzumab had a median DFS not reached if N0 and 114 months if N+ (p 0.297) and a median OS not reached if N0 and of 157 months if N+ (p 0.475), showing differences not statistically significant according to the nodal status. Also patients with ER+/PR±/HER2+ tumors treated with trastuzumab had median DFS and OS not significantly different based on nodal status: median DFS was 95 months if N0 and 85 months if N+ (p 0.615) and median OS was not reached if N0 and of 106 months if N+ (p 0.436).

Patients with ER−/PR−/HER2+ tumors not-treated with adjuvant trastuzumab had a median DFS not reached if N0 and 26 months if N+ (p 0.279) and a median OS not reached if N0 and of 34 months if N+ (p 0.273), showing differences not statistically significant according to the nodal status, but with times to disease recurrence shorter than other subgroups. Patients with ER−/PR−/HER2+ tumors treated with trastuzumab had median DFS significantly different based on nodal status, as median DFS was not reached if N0 and 66 months if N+ (p 0.014), while OS was not reached neither if N0 nor if N+ (p 0.094) (Table 6).

**Table 6:** Median DFS and OS according to nodal status and treatment with adjuvant trastuzumab

		Median DFS (months)	*p*	Median OS (months)	*p*
**ER+/PR±/HER2+ pre-trastuzumab**	**N0**	not reached	0.297	not reached	0.475
	**N+**	114		157	
**ER+/PR±/HER2+ post-trastuzumab**	**N0**	95	0.615	not reached	0.436
	**N+**	85		106	
**ER−/PR−/HER2+ pre-trastuzumab**	**N0**	not reached	0.279	not reached	0.273
	**N+**	26		34	
**ER−/PR−/HER2+ post-trastuzumab**	**N0**	not reached	0.014	not reached	0.094
	**N+**	66		not reached	

## DISCUSSION

In this retrospective analysis, we estimated the role of well established prognostic factors in 1198 early BC patients, over a period of about twenty years. Our findings suggest that nodal status and IHC tumor pattern still represent useful tools in the treatment selection and follow-up decision making of patients in clinical practice. We found a statistically significant difference in both DFS and OS between patients with N0 and N+ tumors (DFS: 165 versus 102 months; OS: 196 versus 134 months), confirming that lymph node negativity is an important predictor of better long-term outcome in early BC: Colzani et al. [6] found that lymph node negativity at diagnosis was the only independent factor associated with favorable prognosis in these patients and reported that, in agreement with a previous study [15], the effect of lymph node positivity on survival was still evident many years after diagnosis, conferring an increased risk of death even after 10 years. More, they did not detect a statistical difference between having one to three or four or more positive lymph nodes among women ≤ 45 years, but, in the overall analyzed population, they showed that the number of positive lymph nodes was related to survival in the first 5 years after diagnosis, while there was a tendency for the curves to converge thereafter. Consistently with these results, in our findings, although differences in survival between patients with N1-3 and N4 tumors were not statistically significant, the 2 y, 5 y and 10 y-DFS rates show that the probabilities of disease recurrence depend on the number of positive lymph nodes more within the first 5 years after diagnosis (2 y-DFS rates: 83.6% if N1-3; 78.3% if N ≥ 4) compared to what happens from 5 years onwards (5 y-DFS rates: 65.2% if N1-3 ; 63% if N ≥ 4; 10y-DFS rates: 46% if N1-3; 42% if N ≥ 4).

Our results show that the greatest impact of lymph node status on survival occurs for the ER+/PR±/HER2−and ER−/PR−/HER2+ subgroups, where differences in DFS and OS are statistically significant between N0 and N+ cases, while for the TN and ER+/PR±/HER2+ subgroups there is a trend toward better prognosis for N0 cases, but not reaching statistical significance. The explanation of the not significant impact of nodal status among TN BC patients should be sought in the biological aggressiveness of this disease, as argued below. Patients with TN BC showed the worst prognosis in our analysis, reporting the shortest DFS and OS, in line with literature data [16–18]. As previously mentioned, in this subgroup, the 1-year and 5-year DFS rates were 86.5% and 60%, respectively, with a plateau in the curve starting from about 100 months from diagnosis (8-year DFS, 40%). This characteristic pattern of recurrence of TN BC has been already described by Dent et al. [19] who reported that, in their study, the risk of recurrence increased rapidly from diagnosis, reached a peak between 1 to 3 years and then decreased quickly. Consistently, Liedtke et al. showed that, after neoadjuvant chemotherapy, recurrence and death rates for TN BC were strongly time-dependent and higher during the first 3 years after diagnosis [20]. The same phenomenon was observed in a more recent study assessing time of recurrence and factors influencing outcome in patients with TN BC [21]: the highest risk of relapse occurred during the first 3 years after primary treatment and then, during the next 2 years of follow-up, it did not change. The 6-year DFS and OS rates were 68 and 62%, respectively, comparable to our findings. Notably, authors found that the tumor size was responsible for recurrence despite lack of involvement of lymph nodes, while nodal status, together with tumor size, adjuvant/neoadjuvant treatment and metastases in the brain, liver and bones influenced OS. In a large cohort of patients with TN BC, evaluating the clinical outcomes and the relationship between tumor size, lymph node status and prognosis, the 5-year OS was 80% for N0 patients, 65% for N1-3, 48% for N4-9, and 44% for N ≥ 10 (*p* < 0.0001); the 5-year RFS rates were 67% for N0, 52% for N1, 36% for N2, and 33% for N3 (*p* < 0.0001). Even if these survival rates are comparable to ours, authors showed that, when comparing N0 with N+ disease, there was a significant difference in OS and DFS. However, once there was evidence of lymph node metastasis, the prognosis could not be affected by the number of positive lymph nodes [22]. In the TN subgroup, we reported a median DFS 109 months and 65 months in case of N0 and N+ tumors, respectively (p 0.144) and median OS 158 months and 96 months, respectively (p 0.384), with not statistically significant differences, but with a trend toward a poorer prognosis in case of nodal involvement. Thus, in our experience, the nodal status seems not to influence the long-term outcome of patients affected by this poor prognosis disease, making us hypothesize that the biological aggressiveness of the disease has a greater impact on prognosis compared to its extension. On the other hand, in the ER+/PR±/HER2- subgroup, given the biological less aggressive behavior of the disease, tumor extension could have a major role on prognosis. In this subgroup, in fact, median DFS was 165 months and 114 months in case of N0 and N+ tumors, respectively (*p* < 0.001) and median OS 166 months and 144 months, respectively (p 0.028), confirming, among other, their overall better outcome compared to the other subgroups [23]. Clearly, bias related to the retrospective data analysis and to the different type of chemotherapy performed could have had an impact on outcome. The lack of a significant impact of nodal status on prognosis of patients with ER+/PR±/HER2+ BC could be due to the effect of endocrine therapy that they received and to its interaction with the intracellular pathway of the HER2 receptor. The hormone-receptor status may influence clinical behavior not only in HER2-negative, but also in HER2-positive BC.

In the HER2+ cohort of our study, we noticed a different prognosis of patients according to hormone-receptor, nodal status (even if significant only for the subgroup of patients with ER−/PR−/HER2+ tumors treated with adjuvant trastuzumab) and treatment with adjuvant trastuzumab, consistent with data of the multicenter observational RETROHER study, where relapse rates at 3 years were higher in case of node-positivity, hormone-receptor negativity and diagnosis in the pre-trastuzumab era [24]. Our data show that, before the introduction of trastuzumab in the therapeutic management, the difference in DFS and OS between hormone-receptor positive and hormone-receptor negative patients is not significant. Anyway, from the survival analysis it is clear that up to 120 months from diagnosis the two curves are separated, while after 120 months they overlap: we hypothesized it may be charged to the carry-over effect of endocrine therapy [25]. It has been recently reported that outcomes in HER2+ patients with early BC not receiving anti-HER2 therapy strongly depend on hormone-receptor expression: in line with our findings, hormone-receptor positive tumors had a slowly decreasing hazard compared to hormone-receptor negative tumors, which showed a higher and more quickly declining hazard of disease-recurrence during follow-up; at about 6 years from diagnosis, the two curves tended to cross [26]. On the other hand, in our series, patients treated with adjuvant trastuzumab showed separated curves with statistically significant differences: trastuzumab deletes the negative prognostic effect of HER2 and the role of hormone-receptor status turns clear. In HER2+ advanced BC, expression of ER, PR or both receptors in ≥ 30% of tumor cells was significantly associated with an improvement in PFS compared with lower or null expression. In this setting, the administration of maintenance endocrine therapy to patients with hormone-receptor positive tumors led to a significant reduction in the risk of progression compared to patients not receiving endocrine therapy. Interestingly, high hormone receptor expression was associated with a non-significant trend toward reduced risk of progression also in patients not receiving maintenance endocrine therapy [27]. It is now widely recognized that HER2+/hormone receptor-positive and HER2+/hormone receptor-negative tumors fall into two distinct subtypes, carrying a different prognosis in the absence of HER2 targeted therapy [28–29] and that this different clinical behavior can be explained by the molecular mechanisms guiding their biology, mainly represented by the bidirectional and dynamic cross-talk between the HER2 and the hormone receptor pathways [30–31]. The different biology between ER+/PR±/HER2+ and ER−/PR−/HER2+ BC could explain the different impact of nodal status on long-term outcome of affected patients. Our results show that median DFS between node negative and positive patients among the ER+/PR±/HER2+ subgroup is not significantly different, contrarily to what happens among the ER−/PR−/HER2+ subgroup. As disease recurrences occur later for ER+/PR±/HER2+ BC, regardless of treatment with anti-HER2 therapy (as previously argued), we hypothesize that patients in this subgroup have not yet fully matured all their relapse events, also considering that they received endocrine therapy, with its abovementioned carry-over effect.

A bias related to the shorter follow-up of patients with HER2-positive BC treated with trastuzumab also explains why, according to Kaplan-Meier curves (Figure 4), the 10-year DFS and OS of ER+/PR±/HER2- BC patients seems worse than ER+/PR±/HER2+. We believe it is due to the fact that, starting from 2006, all recurrences have not yet occurred.

Surely, the present study presents some substantial limitations, mainly related to the retrospective methodology used. Furthermore, in the interpretation of results we also have to consider the heterogeneity of adjuvant treatments delivered, the different follow-up length for the various subgroups (particularly between HER2+ patients treated and not-treated with adjuvant trastuzumab, belonging to sequential and not parallel cohorts) and the small number of patients in some subgroups (especially when the HER2+ cohort was divided according to the hormonal-receptor and nodal status). Finally, the IHC analysis of tumor samples was not centralized, but pulled out from patients’ medical records. In particular, we are aware that chemo- and endocrine therapies have greatly improved over the twenty years of retrospective observation, but they transversally involved all BC subtypes. Thus, we believe that data analysis may still be valid. Objective of this study was not to assess the impact of different treatments on prognosis of our patients, but to evaluate how, in clinical practice, the extension and the biology of disease can help us better define the surveillance strategies. Despite these limitations, the strength of this work lies in the analysis of a large series of patients, all treated according to the standard of care, over a period of about 20 years and then with a follow-up long enough to capture a large number of recurrences.

In conclusion, the main aim of surveillance after the primary treatment of BC is the early detection of disease recurrences potentially treatable with radical intent, as well as the monitoring of long-term effects of therapies. We believe that the concept of personalized medicine is to be applied not only to the therapeutic management of patients, but also to the monitoring phase for an adequate follow-up, differentiating, through validated prognostic models, categories at highest risk which may require a more intensive surveillance than lower-risk categories. Our results could be a useful tool helping physicians in their clinical decision making as well as in the selection of the better follow-up strategies for their patients.

## MATERIALS AND METHODS

### Ethics statement

All procedures performed in this study, involving human participants, have been conducted in accordance with the 1964 Helsinki declaration and its later amendments or comparable ethical standards. Written informed consent was obtained from all patients.

### Methods

Present analysis included patients with early invasive BC treated in the adjuvant setting at Medical Oncology Department, San Salvatore Hospital, L'Aquila, Italy, over a period of about twenty years (from June 1992 to December 2013).

Information on date and age at BC diagnosis, tumor characteristics, local and systemic therapy, date of local or distant disease recurrence, date of last follow-up and date of death were retrieved from patients’ medical records. Staging of primary tumors was based on the TNM pathological cancer staging classification, 7th edition [32]. Tumor stage was defined according to the greatest dimension of the largest tumor size (T1a, ≤ 0.5 cm (including micro-invasion); T1b, > 0.5 cm and ≤1 cm; T1c, > 1 cm and ≤ 2 cm; T2, > 2 cm and ≤ 5 cm; T3, > 5 cm, T4, any size with direct extension to chest wall and/or skin). Lymph node status was described according to the number of regional lymph nodes with pathologically proven metastasis, including results of sentinel lymph node excision. Lymph nodes with only isolated tumor cells were defined lymph node negative (N0, no pathologically proven positive lymph nodes; N1, 1–3 positive, N2, 4–9 positive; N3, ≥ 10 positive). Grading of tumors was based on the AJCC grading system, 7th edition [32]. From 1992 to 1999, the analysis of ER and PR was performed by the immunocytochemical method (Abbott monoclonal ER-ICA and PG-ICA, for ER and PR, respectively); from 2000 to 2013, the analysis was done with the IHC method, using Dako monoclonal antibodies throughout the entire study period: for ER, ER 1D5 Dako antibody was used from 2000 to 2010 and ER EP1 Dako from 2011 to 2013; for PR, Dako PgR 636 was used from 2000 to 2013. Patients were considered ER-positive and PR-positive in case of ≥1% tumor cells nuclear staining at IHC analysis. HER2 analysis was done using the HercepTest (Dako), with staining intensity score evaluated from 0 to 3+. For specimens staining 2+, Fluorescent *in situ* Hybridization analysis was performed to assess HER2 amplification (ratio > 2.2). HER2 status was registered from 1999 and onward. Among enrolled patients, 102 (8.5%) and 1096 (91.5%) received diagnosis of BC before (first generation) and after 1999 (second generation), respectively.

Among patients from the second generation, two cohorts were analyzed: patients who received adjuvant chemotherapy without trastuzumab, mostly until 2005, and patients who received adjuvant chemotherapy followed by or combined with trastuzumab since 2006, when adjuvant trastuzumab was approved in Italy.

### Statistical analysis

Primary end-point of the study was the evaluation of the DFS and the OS of the whole population and according to tumor characteristics.

DFS was defined as the time from diagnosis of BC to time from surgery to any invasive or non-invasive BC recurrence, either local, regional, contralateral or distant; OS was defined as the time from diagnosis of BC to death from any cause or to last follow-up [33].

Kaplan-Meier curves of DFS and OS were compared by the log-rank test, with statistical significance set at *p* ≤ 0.05.

## Abbreviations

BC: breast cancer
HER2: human epidermal growth factor receptor 2
ER: estrogen receptor
PR: progesterone-receptor
DFS: disease-free survival
OS: overall survival
IHC: immunohistochemical
CI: Confidence Interval
HR: Hazard Ratio
TN: triple negative
